# Healthcare service providers’ and facility administrators’ perspectives of the free maternal healthcare services policy in Malindi District, Kenya: a qualitative study

**DOI:** 10.1186/s12978-015-0048-1

**Published:** 2015-06-27

**Authors:** Evaline Lang’at, Lillian Mwanri

**Affiliations:** Discipline of Public Health, School of Health Sciences, Faculty of Nursing, Medicine and Health Sciences, Flinders University, SA, Level 2 Health Sciences Building Registry Road Bedford Park, SA 5042 Flinders, Australia; Ministry of Health, Kenya. Kilifi County Government, GPO Box 9–80108, Kilifi, Kenya

**Keywords:** Free maternal healthcare service policy, Healthcare providers, Facility administrators, Malindi District, Kenya

## Abstract

**Background:**

Globally, there are increasing efforts to improve maternal health outcomes including the reduction in maternal mortality rates. Improved access to skilled care utilisation during pregnancy and delivery has been one of the strategies employed to improve maternal health outcomes. In Kenya, more than half of the women deliver without the assistance of a skilled attendant and this has contributed to high maternal mortality rates. The free maternal healthcare services policy in all public facilities was initiated as a strategy to improve access to skilled care and reduce poor maternal health outcomes. This study aimed to explore the perspectives of the service providers and facility administrators of the free maternal health care service policy that was introduced in Kenya in 2013.

**Methods:**

A qualitative inquiry using semi-structured one-on-one interviews was conducted in Malindi District, Kenya. The participants included maternal health service providers and facility administrators recruited from five different healthcare facilities. Data were analysed using a thematic framework analysis.

**Results:**

Free maternal healthcare service provision was perceived to boost skilled care utilisation during pregnancy and delivery. However, challenges including; delays in the reimbursement of funds by the government to the facilities, stock outs of essential commodities in the facilities to facilitate service provision, increased workload amidst staff shortage and lack of consultation and sensitisation of key stakeholders were perceived as barriers to effective implementation of this policy.

**Conclusion:**

Free maternal healthcare services can be one of the strategies to improve a range of maternal health outcomes. However, the implementation of this policy would be more effective if; the healthcare facilities were upgraded, equipped with adequate supplies, funds and staff; the community are continually sensitized on the importance of seeking skilled care during pregnancy and delivery; and inclusivity and collaboration with other key stakeholders be fostered in addressing poor maternal health outcomes in the country.

## Background

Maternal morbidity and mortality have been a major public health concern across the globe and more so in developing countries. Globally, more than 800 maternal deaths are reported per day with 99 % of these deaths occurring in low and middle income countries [1].

Pregnancy related conditions in Africa have been identified as significant causes of deaths in women between the ages of 15–44 years. A woman in Africa has a 1 in 39 lifetime risk of dying from pregnancy compared to 1 in 4000 in high income countries [2]. Compared to other parts of the world, maternal deaths are higher in Sub-Saharan Africa where approximately 165,000 women are reported to die annually due to pregnancy and its related complications [1].

Kenya is evolving from a low to a middle income nation but despite this economic milestone, Kenya is still ranked among the ten countries that contribute to the 60 % of global maternal mortality – Kenya records over 6000 maternal deaths annually [3]. High costs of maternal health care, especially deliveries, coupled with continuing high maternal mortality rates, have led to a debate about optimum health financing strategies for maternal health, [4] and questioned inequities in health service accessibility brought by user fees [5]. User fee on health care services was introduced in Kenya in 1989 as a health financing strategy [6], however this strategy has since been seen to contribute to inequity in the utilisation of health care services as the poorest households were found to utilise less of the fee charging facilities compared to the rich in the society [7].

It is well recognised that a significant proportion (56 %) of women in Kenya especially those who live in rural settings do not deliver their babies within a health facility or assisted with a skilled birth attendant [8]. For those who deliver at home, 28 % are reported to do so with the help of a Traditional Birth Attendant (TBA), one-fifth deliver with a relative, and nearly one tenth of the women deliver alone [9, 10]. Culture, poverty (including pregnant women’s inability to pay for skilled care services) and distance to a health facility [8] have been recognised as factors that lead to women delivering their babies outside of the health care facilities.

In order to improve this poor situation, the government of Kenya has taken several measures to address issues of poor maternal health outcomes in the country. One of these measures include the introduction of a fee free policy on maternal health services since June 2013 [6].

The fee free policy aims to improve the accessibility, and utilisation of quality maternal and newborn health care across all demographic groups in Kenya as well as reduce the unmet maternal health needs [8]. Additionally, the introduction of free skilled care delivery services intends to improve access to health services for disadvantaged communities, [5] by reducing the financial barrier of accessing skilled care. [4]

The World Health Organisation (WHO) defines skilled care as ‘*quality care to a woman during pregnancy*, *childbirth and postpartum period by a skilled personnel supported by an enabling environment* (*necessary equipment*, *supplies and medicines and infrastructure*) *and a functional referral system* [11]. *At the centre of skilled care is a skilled attendant who is a health professional* (*midwife*, *nurse*, *doctor*) *educated and trained to proficiency in the skills needed to manage normal pregnancy*, *childbirth and the immediate post*-*partum and in the identification and management or referral of complications*.’ [11]

The fee free policy aligns with other international strategies to reduce maternal mortality rates including the Millennium Development Goal 5 (MDG5) that calls for a reduction in maternal mortality by three quarters by 2015. [4, 8, 11].

As far as is known, there have not been any studies that have investigated how the policy stakeholders perceive this policy. This paper is part of the larger study to explore and triangulate the perspective of all stakeholders of this policy. The research sought the views and perspectives of a wide range of individuals who represent service providers, facility administrators and service users from Malindi District. The aim of this paper is to report perspectives of health service providers and facility administrators about some of the factors that act as barriers and facilitators to the implementation and provision of the free skilled maternal health services introduced in Malindi District. Data analysed for this paper are taken from interviews from these selected stakeholders to highlight their understanding and inform practice (policy implementation and service provision) and policy (policy planning and feedback) in order to improve overall maternal health outcomes in the district.

## Methods

### Study setting

The study was conducted in Malindi district, a sub county within Kilifi County in the Coastal parts of Kenya. Malindi district covers an area of 7750 km^2^ and has an estimated population of 424,081people with nearly half (47 %) of the population being below the age of fifteen years [12]. The district is served by 37 government owned health facilities. Thirty six (97 %) of these facilities are equipped to offer Basic Emergency Obstetric Care (BEOC) which includes; antenatal care with early detection and treatment of common problems of pregnancy, as well as first aid for complications of pregnancy and labour. The other facility (Malindi district hospital) is the only government owned facility in the district which is equipped to offer Comprehensive Emergency Obstetric Care (CEOC) to include the provision of BEOC, blood transfusions and caesarean sections [13]. The current Maternal Mortality Ratio in Malindi district (MMR) is at 428 per 100,000 live births [14] with an equally high fertility rate of 6.1 children per woman, higher than the national fertility rate, of 4.6 children per woman [15].

### Theoretical framework

The study espoused the Realist Evaluation Framework introduced by Pawson & Tilley [16].

This theoretical framework identifies and provides the lens through which to understand the best practical method or methods in approaching or causing change whilst giving consideration to the context. Realism begs the question, ‘what works for whom, in what circumstance and in what respects and how?’ [16, 17]. Realism is also said to be an ‘open system’ in that, it acknowledges that the program is prone to being affected by external factors. Therefore, one cannot assume a program to take a constant state but has to acknowledge the fact that a program changes throughout its cycle for it to remain viable [16]. Realism explains the programs failure and/or success [16, 17], enabling the planners to know whether a program can be applied in another setting or not and goes further to give an explanation as to why so or why not. Realism appreciates the fact that the program is active and works around active recipients hence the choice of the evaluation methodology ought to fit in this active arena [16].

Realism not only focuses on the effects of a program but also on the inner workings and operations of a program and how they are connected, which is often obscured from those who observe the outcome patterns. These workings and operations have been termed as the Context Mechanism Outcome (CMO) configuration. Mechanism forms the pivot point in realist evaluation [17]. Context on the other hand is not only linked to the place but also the systems, relationships, biology, technology and economic status. Context elements are those factors that are external to the intervention, present or occurring even if the intervention does not lead to an outcome, and which may have an influence on the outcome. It describes those features or the conditions in which programs are introduced that are relevant to the operation the program mechanisms. Mechanism is built on the premise that it is not the program that works but the action elicited by the target audience or stakeholders equipped with the right resources and capabilities offered by the program is what makes the program work [16].

A realist approach looks for mechanisms at individual, group, organizational and societal levels .In the case of the current study the mechanism is the actions or change elicited and experienced by the, providers and facility administrators following the elimination of user fee on maternal health services.

Applying this evaluation framework, the study aims at identifying what works for whom, in what circumstance and in what respects and how, with reference to the user fee elimination program on maternal health services in Malindi district.

### Data collection

A qualitative inquiry was employed to explore the perceived impact of free skilled maternal healthcare service provision in Malindi district. Purposive sampling was used to recruit the study participants. The inclusion criteria for the study was that one had to be a health service providers (nurses, doctors, and consultants) or a facility administrator (facility in-charges and hospital administrators) who has served in Malindi District for at least two years or more. Fifty introduction letters requesting their participation in the study were sent out to the potential participants’ offices, departments and stations within Malindi District. The letter clearly described the study’s aim, and informed the potential participants that their interest to take part in the study was absolutely voluntary and there was no consequence if they choose not to participate. The letter also bore the researcher’s telephone contacts where the potential participants were to send a text message to the researcher upon their acceptance to participate in the study. The potential participants were informed that the study was to be conducted in an interview format, and the interview would take approximately one hour.

Of the fifty introduction letters sent out, 18 potential participants sent text messages to the researcher indicating their interest to take part in the study. Information sheets and consent forms were sent to the 18 participants together with a letter inviting them for an interview to be held at their health facility. The participants returned the signed informed consent form on the interview day. An interview was scheduled with each potential participant at a time and date favourable to them in order to ensure that the service provision was not disrupted in any way. The participants were assured the information they provide was and would remain anonymous. To preserve and protect confidentiality of the participants, each participant was assigned a unique Study Identification Number (SID). At the completion of the study, the identification numbers were de-linked from personal identifiers. This procedure ensured that no data was linked back to an individual.

Semi structured interview guides [18] were developed by researchers and pretested on non-participating district service providers and administrators. Eighteen individual interviews were undertaken with study participants from Malindi District hospital, Kakoneni dispensary, Watamu dispensary, Ganda dispensary and Gede health centre between January and March 2014. An iterative approach was adopted, in that the forthcoming interviews were informed by what arose out of the data collected [19]. The interviews were conducted in English, digitally recorded (with consent of participants) and transcribed by the principal researcher (EL).

Some of the topics that were addressed in the interview guide included “the views, opinions, attitudes and experiences of the participants on the fee free maternity services; the barriers and facilitators of the fee free maternity services and; their commendation and recommendation on the service following the introduction of the policy.”

### Data analysis

All transcripts were repeatedly read by both authors, and subsequently independently analysed thematically using ‘Framework analysis by Ritchie and Spencer [20]. Framework analysis make use of systematic method to manage data giving qualitative data consistency and structure [21]. To minimize the possibility of losing relevant themes, repeated themes of text were identified, allocated headings conferring to the context and coded to numerous important categories. Sub-headings were then identified from the thematic analysis [21]. This method leads to a greater transparency, rigor and validity of the process of systematic analysis. Analysis was both deductive, with categories derived from prior knowledge, and inductive, with categories emerging purely from the data [22].

Triangulation of narratives of different individuals at different levels of health system, and different settings contributed to the validity of the data and highlighted any conflicting views and widely shared themes across the group.

### Ethical consideration

Ethical approval was obtained from the Social and Behavioural Science Research Ethics Committee, Flinders University (approval number 6331) and formal permissions obtained from the office of the Director of Health Kilifi County (Ref no: ST.HP/KRCHS/VOL.II/152).

Data were collected once the participants had signed an informed consent. The participants were also informed that their participation was voluntary and they could withdraw from the study during the interview if they so wished [23, 24]. Anonymity of participants was maintained including through one-on-one interviews. No incentive was given.

## Results

### Healthcare service providers

Twelve healthcare service providers were recruited (Table 1). Ten of them were midwives working in different departments within the reproductive health units in all the five facilities. Of these midwives, five work in Malindi district hospital and two work in Ganda dispensary. Gede health centre, Watamu dispensary and Kakoneni dispensary had one midwife each. The two additional service providers included a consultant obstetrician/gynaecologist and a medical officer who work in the reproductive health unit.

**Table 1:** Characteristics of Service Providers

Type of health care worker		Department where currently employed		Work experience in years		
Type	N	Area of work	N = 12	0-4	5 -9	>9
Midwives	4	Maternity department	4	3	0	1
Midwives	6	Antenatal and postnatal unit	6	0	0	6
Consultants	1	Reproductive health unit	1	0	1	0
Medical officers	1	Reproductive health un	1	1	0	0

#### Healthcare facility administrators

Six administrators were recruited four of whom were Nursing Officer In-charges and two were facility administrators. Table 2 shows the range of facility administrators involved in the study and the number of years they have worked.

**Table 2:** Characteristics of Facility Administrators

Type of facility administrators		Work experience in years		
Type	N	0-4	5 -9	>9
Nurse in charge	4	1	2	2
Hospital administrators	2	1	1	0
Total	6	2	3	2

### Themes

Three emergent and often inter-linking themes were identified from the narratives obtained from both the service providers and the facility administrators. These included: (i) perceived benefits of free maternal health services by service providers and administrators, (ii) perceived barriers and/challenges to free maternal health care service policy, and (iii) future management of free maternal services in Malindi District.

### Perceived benefits of free maternal health services by service providers and administrators

Before the introduction of this policy, the maternity departments were overcrowded with mothers who overstayed in the facility due to pending hospital bills. This was reported as a significant cause of stress to health care providers. The free service policy has alleviated this problem as women can go home immediately upon their discharge.

*‘Overall I can say that free maternity service is doing a good thing for example, we used to have so many mothers crowded in the maternity ward because they were waiting to clear the bills but nowadays, they deliver and go home depending on the mode of deliver, if normal delivery they stay in the facility for 24 hrs, if caesarean section we discharge them on the 3*^*rd*^*post-operative day.so we never have mothers overcrowded in the department like we used to. This has eased the pressure on the health care workers’ (IDI with service provider 2).*

The health care providers also perceived the policy as addressing the use of skilled care during pregnancy and delivery in Malindi district. One service provider stated that women who never before visited the facility during pregnancy and delivery as a result of the user fee were now able to seek skilled care services.

*‘Free maternity services will be a solution to the problems in maternal health, for example, the mothers who never used to come to the hospital because of the funds are now coming to the hospital,’ service provider 5.*

The service providers realised a rise in the number of antenatal care services visits as well as number of facility based deliveries following the introduction of the policy.

*‘Another difference I have seen after the free maternity service is that many people are coming to deliver at the hospital as compared to the previous time they could wonder where are they are going to get the money to deliver at the hospital, so they preferred delivering at home. So now I can say that the turn up is big and is good’. Service provider 4*

*‘With the free maternity service, the mothers in the clinic have really increased, like right now, the antenatal care clients are still coming for the service but when you compare this numbers in maternal and child health clinic and those in maternity, they are not tallying so that means that yes we are giving the services in the antenatal clinic, but when you look at the maternity despite the increase in deliveries, their numbers are still few’ service provider 6*

Other service providers stated that the free maternal health care service had already impacted the maternal health outcomes including a reduction in the number of maternal mortalities following the implementation of the free maternity service:

*‘The outcome I can say it is good, eh, the mortality has gone down, because there are times when we had many mortalities, although it was mainly caused by referrals from the rural health facilities, but I can say that after the free maternity service, we have not been having many mortalities,’ service provider 2.*

*‘The maternal deaths are very few, we can stay even up to three months without a maternal death,’ service provider 1.*

The timing of the women seeking these services had also improved. Following the inception of the policy, women were seeking maternal health service sooner than they used to. However, although there was an increase in the uptake of some services, other services were not necessarily accessed. This included the family planning services where the uptake was stated to be very low despite the services being free.

*‘The numbers of pregnant women attending the antenatal visits have increased but the uptake of family planning is very low. The future of our community is good with this free maternity service, however the success lies on preventing these pregnancies as well,’ consultant.*

### Barriers and/challenges to free maternal health care services policy in Malindi.

Despite the positive outcomes of the policy significant challenges and barriers in its implementation existed. These included; “uncompensated loss in fee revenue while patient volumes simultaneously increased”, “inadequate human resource”, “shortages of commodities”, and “demotivation of health care providers”. For example, failure of the national Government to reimburse the facilities promptly was reported to contribute to shortage of drugs and supplies, which negatively affected the quality of care provided. One service provider stated that they were at times forced to improvise or ask the relatives to buy the necessary supplies in order to facilitate service provision in public health care facilities.

*‘Free maternity service is very hectic for the midwives and mothers because when the mother comes to the facility usually we don’t have the supplies and the maternity is usually very bare so we go to some facilities to borrow some supplies.at times we are forced to improvise in order to provide care. We do not get some uterotonics drugs, we do not have delivery packs, and the supply is really low. It is very hectic.’ Service provider 4*

The uncompensated loss in revenue was perceived as a source of frustrations to the administrators who feared that the continuity of service provision may be jeopardised if this challenge persisted. The lack of supplies, drugs and equipment was not only viewed to affect the facility administrators and health care workers, but was also seen to be affecting the other parallel programs that aimed to improve the utilisation of service that had skilled care. For example, TBAs were being given a token for every mother they brought to the facility to deliver. The small token seemed to encourage the TBAs to bring the mothers to the facility instead of conducting the deliveries at home. This initiative seemed to have strengthened health facility’s linkages with the TBAs. As a result, there was improvement in the uptake of delivery services in a facility resourced with skilled attendant. However this has since stopped due to failure to sustain the program soon after the introduction of free maternity services.

*‘We had a meeting with the community to sensitize them on free maternity services and the numbers really increased but we have been having challenges with the loss of revenue because previously we had an arrangement as a facility to reimburse the TBAs when they bring the mothers but now since we have not been having the funds to support this move the TBAs have not been bringing the mothers.’ Service provider 1*

Failure of the government to involve health providers and administrators in the development of this policy was also perceived as barriers to effective implementation of the program in specific facilities.

*‘There was no form of involvement before the rolling out of the program. I think this had an effect in the implementation because if we were involved for example as a medical officer probably we would have talked of the areas that would have been of emphasis like planning of it, and probably we would have ideas on how to implement it well.’ (IDI with a Medical officer)*

On the other hand, facility in charges expressed concerns about the quality of the services provided which they felt was compromised by the high patient volume amidst shortage of staff and supplies.

*‘Free maternity service is a good program, we are happy it is there but the only problem is that it’s quite hectic for us because of the hardships we are facing and, it’s quite hectic for us because we are not offering that quality service that we want to give. Workload has also increased and staff remain constant. This has been a very big challenge hence you end up not giving the quality service due to burnout,’ facility in charge 1.*

The consultant and one service provider stated that sometimes the outcomes are not very good not because the service provider was not there but because the number of women needing a particular service at a particular time significantly outnumbered the service providers.

*‘Sometimes outcomes are not very good for example one nurse who has to deliver 5 mothers cannot monitor 5 mothers all in labour,’ consultant.*

*‘Because of the shortage of staff at times the clients overwork the service providers and maybe complication could occur not because the mother did not come to the facility early but because the midwives have so many mothers to attend to.’ Service provider 2*

As a result of poor quality of the services and the shortage of supplies and drugs, it was also noted that the number of women seeking the service was diminishing over time. This was experienced a few months after the initial implementation of the program.

*‘When it started, the first few nights we got very many mothers coming to the hospital, like the first 3 days we were delivering up to 17 at night but with time it started going down and this is because in our facilities especially at night of which you know many mothers deliver at night we have only 2 midwives in maternity and 1 medical officer intern, so when they come 17 of them you can imagine what would happen…’ Facility Administrator 2*

Apart from the concerns in the quality of the services provided, the medical officers made an observation that the motivation and morale of the service providers was declining. This was perceived to be attributed to the increase in workload amidst shortage of staff.

*‘There is also the challenge of personnel which is highly affected by the workload and you will find that the people offering the service are few and I have noticed lack of motivation on the part of the health care workers that is general in the entire hospital,’ medical officer.*

Prior to the introduction of the fee free policy, the health care system had just been devolved from the national government to the county government. This meant that budgeting for health care and decision making functions were to be conducted at the County/regional level. The devolution was perceived as a challenge to the implementation of the free maternity health services due to a number of administrative and logistical challenges including those related to the transfer of funds from national government, inadequate funding for implementation of the free maternity services and lack of a clear procurement plan for the purchase of medical supplies.

*‘The free maternity started when we had not devolved health services to the counties, so we don’t know whether the running will be left to the counties or the central government. We are now wondering that now that health services were devolved was this project also devolved, what exactly happened. There is no any feedback that we are getting to say that this is the way forward,’ facility administrator 1.*

Poor referral system and operation time for facilities serving rural areas seemed to have affected the implementation of the free maternal health care services in Malindi District as well. For example, concerns on delays in referring mothers for further management and emergencies from the rural facilities to the district hospital were reported to be potential challenges to the reduction of maternal mortality and morbidity in Malindi. The existence of only one ambulance serving 36 facilities within Malindi district seemed to be the main contributor to the weak referral system.

*‘We also have a challenge in the referral system hence handling emergencies is hard and the referral needs you to be an accompanying nurse and if you are alone it is a challenge hence you are forced to send the mothers with their own transport. Also the ambulance is a challenge as we only have one ambulance in the whole district serving the rural areas and hence it delays emergencies’ service provider 9*

*‘We do not get any mortality because if we get any complication we just refer but usually the line of referral is delayed for up to 5 hours,’ service provider 4*

Most dispensaries in Kenya (the main facilities serving rural areas) are poorly staffed and lack adequately trained skilled workers. This limits the functioning of the facilities, including the operation times of the facilities which was perceived as a challenge to maternity service provision and utilisation.

*‘We also have a challenge where we don’t open at night due to insufficient human resource and naturally most deliveries occur at night. This issue has led many women to still deliver at home.’ service provider 9*

### Future management of free maternal health care services in Malindi

In addition to airing their success and challenges, participants proposed strategies that could improve the implementation of free maternal health services in Kenya. These included:

Sensitising the general public on both the user fee removal policy and the importance of seeking skilled care during delivery.

*‘We need to sensitize the community on why free maternity services. we also need to sensitize the community on why deliver at the hospital, let them know about the high numbers of maternal mortality and why we need to fight for the mothers to stay alive, a home without a mother is nothing, this task is beyond the health care worker only’ facility in charge 2.*

Motivating the health care workers through increased remunerations and sponsorship to trainings programs.

*‘There is a need to have something for the service providers because most programs come with the view of the community and the clients but nothing for the service providers.’ Service provider 7*

*‘The free service is for the mothers, but even the midwives should get some form of motivation. Despite the workload and shortage, they will get motivated, for example, through sponsorship to school and trainings. The health care workers should also get good salaries. If they give us good salaries and they motivate us, we will work.’ Service provider 1*

Improving drugs and supplies in the facilities in order to cope and address increased service utilisation.

Effectively communicating with health workers and managers about the policy vision, goals and activities required for effective implementation of the policy.

*There should also be a link between the top level and the people in the periphery to be able to understand what is happening for a successful implementation of the free maternity service,’ service provider 6.*

Increasing human resource for health to facilities in order to address workload issues.

*‘Our plea is to the government to give us necessary equipment and staff so that we may be able to give quality services, so if we will get everything we are good to go.’ Service provider 1*

Involving the community especially through working with TBAs in order to enhance the uptake of the skilled health care.

*‘With our community they rely so much with the TBA. We need to collaborate with the TBA, and you will see the mothers will come to the clinic for ANC and for the deliveries.’ Service provider 4*

## Discussion

Several studies conducted across the world on the elimination of user fee policy on maternal health services have supported this policy as it does increase the usage of skilled care during pregnancy and delivery [6, 25–30]. They also acknowledge that barriers still exist and if not addressed could compromise the success of this policy. The current study further demonstrates that the elimination of user fee is beneficial notably to the financially disadvantaged populations where it was seen to increase their access to skilled care during pregnancy and delivery. These findings are supported by a number of studies that have documented success on user fee removal in low and middle income countries. The successes documented are; an increase in use of these services especially in poor and uneducated women, [31] an increase in the number of institutional deliveries, [26, 27] an increase in elective caesarean section rates [4, 28], and an increase in antenatal visits [29].

Despite the positive outcomes of the program, the health care workers and administrators experienced challenges in the implementation of the program. The main challenges aired were; uncompensated loss in fee revenue while patient volumes simultaneously increased, high patient volume and inadequate human resource, shortages of inputs like drugs and supplies, a sense of demotivation among the health care providers which was attributed to the increase in workload on the few staff, and compromise to the quality of the service provided. These challenges were not only unique to Malindi but were seen to have affected other areas where the free maternity policy and program has been initiated. In Ghana for example, [32] delayed reimbursement by the government to cater for the loss of user fee revenue at health facilities following initiation of free maternity services led to stock-outs of drugs and supplies, which negatively affected the quality of care provided and resulted in some facilities reinstituting user fees. This was also reported in Burundi where the lack of preparation for the new policy resulted in critical negative consequences for healthcare providers, including stock- outs of drugs, reduced quality of services, disruption of the referral system, and reduced motivation of health workers [33].

The health care workers also expressed their concerns and dissatisfaction in the quality of the services provided which they felt was compromised by the high patient volume amidst shortage of staff. The poor quality of free services was perceived to affect the maternal health outcomes and the consumers’ perception towards the services. For instance, it was noted that the number of women seeking the service had reduced and stabilised at a figure slightly above what was there prior to the initiation of the program. This was experienced only a few months after the implementation of the program. According to Thaddeus and Maine, quality of service is one other factor considered by service users prior to seeking care [34]. Hatts’ standard economic theory on user fee elimination policy further states that, if the interaction between price and the quality of services provided is complicated by fewer revenues to purchase key inputs like drugs and supplies coupled with a short staff, then the quality of the health services on offer may change as the price drops. And if both quality and the price drop simultaneously, the effects on quantity demanded are unknown [25, 30].

The health providers participating in this study observed decrease in staff morale and performance following the implementation of free maternity service which they attributed to the increase in workload amidst having shortage of staff. Demotivation of staff was also reported in Nigeria, where the health workers complained of increase in workload yet there was no increase in remuneration or the number of health workers [30].

The devolution of the health care services was perceived to affect the implementation of the free maternity service. The devolution of health care services was understood to lack clear cut procedures for ensuring adequate oversight on the funding, procurement of medical supplies for the health services, and the delivery of these services. This gave rise to administrative and logistical challenges to the provision of the free service. [35]

In addition, weak referral system also posed as a challenge to the program where lack of adequate ambulatory facilities was said to be the major challenge facing the district. Without adequate and proper interventions the weak referral system could pose as a potential challenge to maternal health outcomes. Thaddeus and Maine, [34] have argued that not getting adequate care in time is an overwhelming reason why women die in low and middle income countries. It was also found that the facilities serving the rural areas, mostly the dispensaries, were only operational during the day despite the fact that most deliveries are known to occur at night. As such it was perceived that the women in Malindi district do not fully enjoy the benefits associated with the user fee elimination policy.

According to Thaddeus and Maine three delay model [34], it is plausible to argue that the main challenges facing free maternal health care service policy in Malindi revolve around the third delay, which is the delay in receiving adequate and appropriate care once in the facility. Such inadequacies may be characterized by shortages in supplies, equipment and lack of trained personnel, incompetence of the available staff, demotivated staff or uncoordinated emergency services.

To address the above challenges, several strategies were proposed by the participants. This included: (i) the need to sensitize the general public on both user fee removal policy and importance of seeking skilled care during delivery. This can be done through public information campaigns and meetings with village leaders to communicate the policy vision and goals to the general public including finer details of what users can expect to experience at facilities; (ii) the need to plan for adequate drugs and supplies to cope with increased utilisation, and plan how to tackle wider drug and supplies problems in the longer term; and (iii) the need to motivate the health care workers as a strategy in achieving successful implementation of the program through sponsorship to school and trainings and giving good remunerations to the health care workers. The health care providers serve as the first point of contact of patients in the health facility, as such, if they are not rewarded and motivated the end result will be patients complaining of being mistreated. This has been documented to be one of the barriers to hospital utilization [36]. Another important aspect is the need to involve and communicate clearly with health workers and managers about the policy vision and goals, as well as about what and when actions will be taken [25, 36]. It is recognized that leadership, supervision, information dissemination and communication are major mediators and moderators of the quality and effectiveness of health care [37]. It is also important to increase human resource strength in the facilities in order to address workload issues and improve quality of services that will attract and retain the high number of mothers seeking skilled delivery following the initiation of the program.

As narrated by some study participants (see service provider 1&4), the need to involve the community in order to enhance the uptake of the program by the community through forming linkages with the TBAs and the community health workers is necessary. It has been recognised that community participation is fundamental in the success of any public health intervention [38, 39] and so would be in the reduction of maternal mortality. The engagement of the women (women of child bearing age, mothers, grandmothers and mother-in-laws) in their health decision making at the grassroots level ensures that their concerns and expectations are addressed, their physical, mental, psychosocial wellbeing are protected and above all their health outcomes before, during and after pregnancy and childbirth are secured thereby reducing maternal mortality [40]. Community health workers are commonly known as the engines of health systems in much of the developing world, and are a tremendous asset, as they connect the patient to the system. They are often the patient’s first point of contact, and play an important role in diagnosing, counseling and triaging what level of facility a patient should be sent. With incentives and proper training, they can counsel a pregnant woman to go for her antenatal care visits, ensure that she gets proper nutrition and is tested and treated for HIV, and help her arrange transportation prior to delivery at a health care facility by a skilled practitioner. All these are important steps in preventing newborn and maternal deaths. [41]

### Strengths and limitations

The use of a qualitative approach in this study provided an in-depth and highly contextualized information and insights into pertinent issues including those that may affect policies and programs. It also covered a wide range of settings involving five facilities within Malindi district that varied in their catchment area, location and level of care which gives an in-depth understanding of events in the program. The study limitation includes the study’s inability to offer a full and complete picture of the user fee elimination policy and program as it was conducted early in the implementation phase of the policy and only 18 interviewers were included to represent service providers and facility administrators. However the useful information gathered from this study could improve the program at this phase and make the later phases a success. Further studies are recommended to evaluate the policy and its implementation at a later stage so as to identify the long term effects on maternal health utilisation, outcomes and service provision.

## Conclusion

The policy on free maternal health services in Kenya aims to improve access to skilled care by all women, especially the disadvantaged rural populations. Consistent with national and international literature, the current study supports the notion that the elimination of user fee increases the uptake of skilled care by the women and has the potential to improve maternal health outcomes in Malindi district [25] if the currently identified challenges are addressed. The findings from the current study provide useful evidence for policy makers in gaining an understanding on what works and what does not work for the program hence enabling them to concentrate efforts to increase the delivery of what we know can be effective in reducing poor maternal health outcomes and accelerating the progress towards the attainment of MDG5.
